# Effects of Galvanic Vestibular Stimulation on Vestibular Compensation in Unilaterally Labyrinthectomized Mice

**DOI:** 10.3389/fneur.2021.736849

**Published:** 2021-09-03

**Authors:** Gi-Sung Nam, Thanh Tin Nguyen, Jin-Ju Kang, Gyu Cheol Han, Sun-Young Oh

**Affiliations:** ^1^Jeonbuk National University College of Medicine, Jeonju, South Korea; ^2^Department of Otorhinolaryngology-Head and Neck Surgery, Chosun University College of Medicine, Gwangju, South Korea; ^3^Department of Neurology, Jeonbuk National University Hospital & School of Medicine, Jeonju, South Korea; ^4^Department of Pharmacology, Hue University of Medicine and Pharmacy, Hue University, Hue, Vietnam; ^5^Research Institute of Clinical Medicine of Jeonbuk National University-Jeonbuk National University Hospital, Jeonju, South Korea; ^6^Department of Otolaryngology-Head and Neck Surgery, Graduate School of Medicine, Gachon University of Medicine and Science, Incheon, South Korea

**Keywords:** vestibular, labyrinthectomy, unilateral labyrinthectomy, galvanic vestibular stimulation, functional recovery, vestibulo-ocular reflex, VOR gain

## Abstract

**Objectives:** To investigate the ameliorating effects of sinusoidal galvanic vestibular stimulation (GVS) on vestibular compensation from unilateral vestibular deafferentation (UVD) using a mouse model of unilateral labyrinthectomy (UL).

**Methods:** Sixteen male C57BL/6 mice were allocated into two groups that comprise UL groups with GVS (GVS group, *n* = 9) and without GVS intervention (non-GVS group, *n* = 7). In the experimental groups, we assessed vestibulo-ocular reflex (VOR) recovery before (baseline) and at 3, 7, and 14 days after surgical unilateral labyrinthectomy. In the GVS group, stimulation was applied for 30 min daily from postoperative days (PODs) 0–4 via electrodes inserted subcutaneously next to both bony labyrinths.

**Results:** Locomotion and VOR were significantly impaired in the non-GVS group compared to baseline. The mean VOR gain of the non-GVS group was attenuated to 0.23 at POD 3 and recovered continuously to the value of 0.54 at POD 14, but did not reach the baseline values at any frequency. GVS intervention significantly accelerated recovery of locomotion, as assessed by the amount of circling and total path length in the open field tasks compared to the non-GVS groups on PODs 3 (*p* < 0.001 in both amount of circling and total path length) and 7 (*p* < 0.01 in amount of circling and *p* < 0.001 in total path length, Mann–Whitney *U*-test). GVS also significantly improved VOR gain compared to the non-GVS groups at PODs 3 (*p* < 0.001), 7 (*p* < 0.001), and 14 (*p* < 0.001, independent *t*-tests) during sinusoidal rotations. In addition, the recovery of the phase responses and asymmetry of the VOR was significantly better in the GVS group than in the non-GVS group until 2 weeks after UVD (phase, *p* = 0.001; symmetry, *p* < 0.001 at POD 14).

**Conclusion:** Recoveries for UVD-induced locomotion and VOR deficits were accelerated by an early intervention with GVS, which implies that GVS has the potential to improve vestibular compensation in patients with acute unilateral vestibular failure.

## Introduction

Galvanic vestibular stimulation (GVS) delivers electrical currents to the vestibular end organs and their afferents, resulting in simultaneous increases and decreases of discharge in the vestibular afferents by cathodal and anodal stimulation, respectively (1, 2). It has been assumed that weak GVS current likely stimulates all primary vestibular afferents acting upon the post-synaptic spike trigger zone by modulating the regularity of the firing rate of vestibular afferents (1, 3, 4), rather than by inducing membrane depolarization of vestibular sensory organs (5–7). Accordingly, the thin and slower-conducting regularly discharging afferents respond symmetrically to the two polarities of GVS, whereas the thick and fast-conducting irregularly discharging afferents show an asymmetrical response with a higher galvanic sensitivity during cathodal stimulation (excitatory) than anodal stimulation (inhibitory) (4, 5, 7–9). Recently, the application of short-term noisy GVS to improve static and dynamic vestibular deficits in unilateral labyrinthectomized rats has been reported (10). Another animal study with monkeys directly investigated single vestibular afferents and showed robust and parallel activation of both canal and otolith afferents in response to GVS (7). On the other hand, some electrophysiological studies on isolated frog posterior semicircular canal (SCC) preparations showed that this electrical stimulation directly modulates neurotransmitter (GABA) release by hair cells (8, 11). The electrical stimulation also contributes to the upregulation of c-Fos protein, which is generally considered as a marker of stress and neuronal activation, in the vestibular nuclei (VN) (12, 13). These findings indicate that GVS can activate vestibular hair cells and the neurons in the VN in the central vestibular pathways.

Clinically, GVS has been used for over 100 years to investigate the role of vestibular signals in gaze, posture, and locomotor control under pathophysiological conditions in peripheral and central vestibular or neurodegenerative disorders as a relatively pure vestibular stimulator (6, 10, 14–18). Recently, GVS has been promoted as a potential therapeutic (18) and diagnostic tool (19, 20) for bilateral vestibular dysfunction. It has been suggested that the plausible mechanism underlying the postural enhancement is the addition of an optimal level of noise into a non-linear system, which can enhance the detection of sub-threshold signals and the processing of information (21, 22). Furthermore, noisy GVS improves postural sway and dynamic gait stability, and subsequently reduces the fall risk in patients with bilateral vestibulopathy (23, 24). Another study also showed that sub-threshold noisy GVS enhanced the vestibulo-ocular reflex (VOR) with increasing amplitudes of ocular vestibular-evoked myogenic potentials in healthy subjects (25).

Despite the recent popularity of GVS for the assessment and treatment of various vestibular disorders, how this non-invasive electrical stimulation recovers the vestibular function in unilateral vestibular deafferentation (UVD) remains uncertain (7, 10). The present study was designed to determine the efficacy of GVS for vestibular compensation, especially on VOR recovery, using a mouse model of unilateral labyrinthectomy (UL).

## Materials and Methods

### Animals

Sixteen male C57BL/6 mice aged 9 weeks and weighing 20–25 g (Animal Technology, Koatech, Kyonggi-Do, Korea) were randomly assigned to two experimental groups: UL with GVS intervention (GVS group, *n* = 9) and UL without GVS intervention (non-GVS group, *n* = 7). Every effort was made to minimize both the number and the suffering of mice used in the experiment. Mice were acclimatized to laboratory conditions for 1 week before the experiment started, then housed separately and kept in a temperature- and humidity-controlled room with free access to food and water.

The animal procedures included in this study were consistent with the Assessment and Accreditation of Laboratory Animal Care International and have been reviewed and approved by the Animal Care Committee of the Gachon University of Medicine and Science (IRB MRI 2019-0008).

### Surgical Procedure for Unilateral Labyrinthectomy (UL)

Both GVS and non-GVS groups underwent right-sided UL. We used surgical labyrinthectomy, which is relatively simple and reliable, induces vestibular symptoms immediately after surgery, and has a faster recovery than vestibular neurectomy or chemical labyrinthectomy (26–28). UL was carried out according to a surgery protocol described previously (29–31). A 10-mm-long skin incision was made 5 mm behind the right auricular sulcus to expose the bony labyrinth, and the muscle and soft tissues covering the temporal bone were dissected. After approaching the horizontal and posterior SCC, a small hole was made in the posterior SCC with a diamond otologic drill (0.5 mm in diameter) for perilymph leakage. Gentle suction was used to aspirate perilymph fluid for 3 min, then the hole was filled with collagen (Helitene; Intergra Life Sciences Co., Princeton, NJ, USA) to prevent further leakage. The skin was closed using a 5–0 Vicryl suture in two layers. The appearance of spontaneous nystagmus, postural asymmetry, and head tilt after recovery from anesthesia confirmed the successful UL. All treated mice were anesthetized by continuous inhalation of isoflurane gas (Ifran, O_2_ 5 L/min, 2.0; Hana Pharm Co. Ltd., Kyonggi-Do, Korea) during surgery as well as in preparation for GVS application.

### Vestibulo-Ocular Reflex (VOR) Recording and GVS Application

A permanent head implant to restrain the animal's head during VOR recording was constructed. After making a small incision in the mouse's scalp, we fixed a small metal plate with a screw hole to the center of the skull using dental cement. A marker (400 um, white isosceles triangle) was attached to the center of the right cornea after local anesthesia to track eyeball movement (32) and then to record ocular movements during VOR stimulation. The left eyelid was covered with ophthalmic ointment (Tarivid® Santen Pharmaceutical Co., Ltd, Osaka, Japan) during VOR recordings to prevent visual input to the other eye. All experimental mice were anesthetized with isoflurane inhalation (Ifran, 2.0%, O_2_ 5 L/min; Hana Pharm. Co.) under sterile conditions.

**Figure 1:**
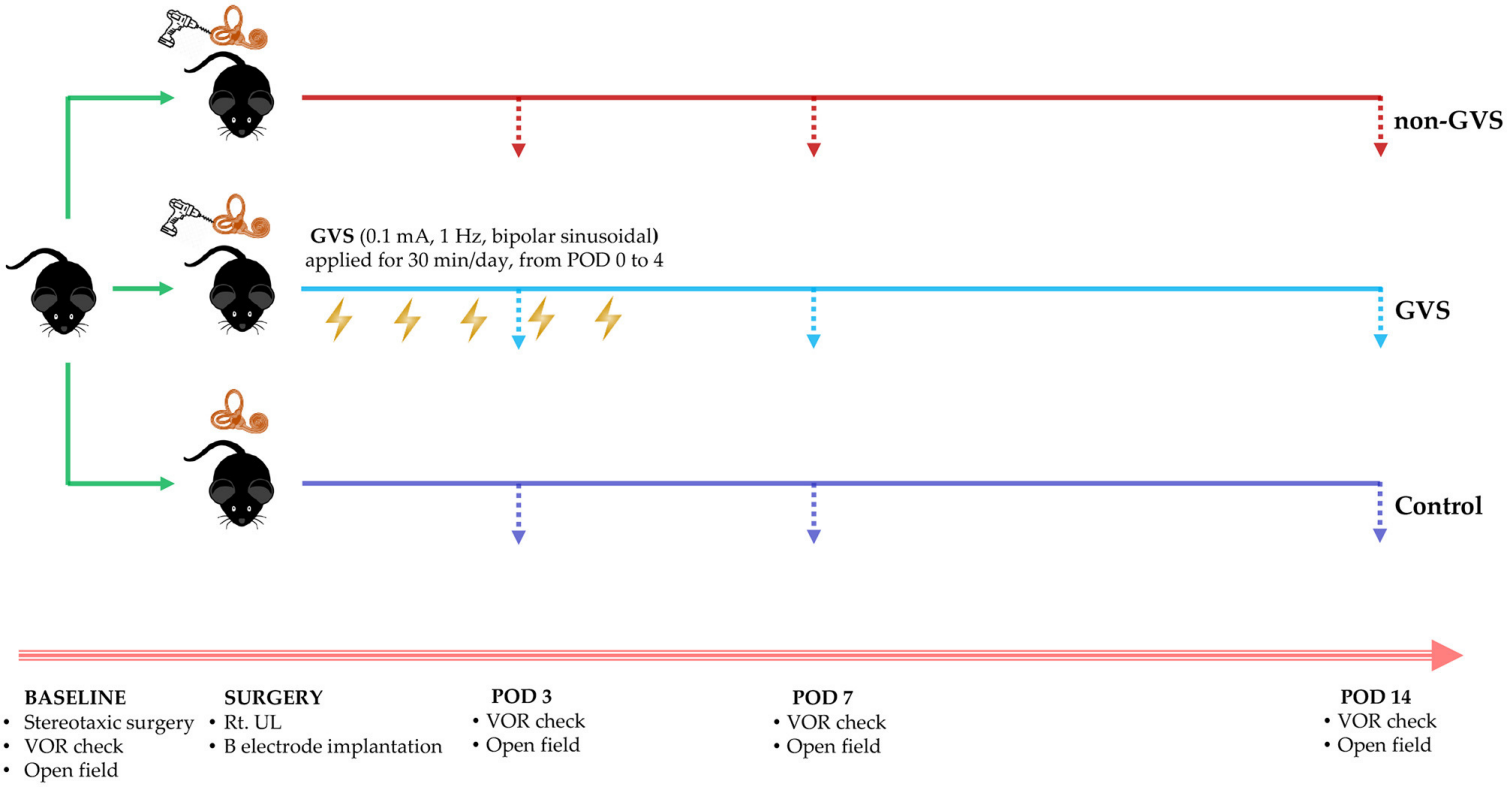
A schematic representation of the experimental design and time scales for GVS application. GVS, galvanic vestibular stimulation; VOR, vestibulo-ocular reflex; UL, unilateral labyrinthectomy.

To measure the VOR elicited by sinusoidal stimulation, the mouse was placed in a cylindrical restrainer that connected to the vestibular turntable. The mouse's head was fixed to the restrainers using the previously implanted head fixation in the scalp. The horizontal SCC was oriented to the earth's horizontal plane, which corresponded to the plane of sinusoidal stimulation. The mouse would be relaxed 40 min before VOR recording for limiting any effects of the anesthesia. The sinusoidal stimulation was delivered by turntable in the horizontal plane at a frequency of 0.08–1 Hz and peak velocity 150°/s (32). VOR measurements were conducted in each group on PODs 3, 7, and 14 (Figure 1).

For the application of GVS, we inserted electrodes made of a metal bolt cap (1.26 mm in diameter) into circular plastic buttons (5 mm in diameter), and then each electrode was attached to one uninsulated tail of a 3-cm-long wire (30G) passing through the skin, which was connected to the galvanic stimulator via alligator connectors. After the implantation of these electrodes near the bony labyrinths, the wound was closed with a 5–0 Vicryl suture to support the healing process. The sinusoidal current was generated by a computer-controlled stimulator with the cathode (excitatory) in the right (lesioned) side and the anode (inhibitory) (33) in the left (intact) side of the mouse. During the pilot experiment, we determined the GVS threshold before the intervention session by delivering a sinusoidal GVS current with 1 Hz, progressively increasing intensity from zero. The GVS threshold, which exhibits a vestibular-specific effect, was the lowest level that evoked a clearly repeatable vertical-torsional nystagmus without any other muscle activity (Supplementary Table 1) (5, 10, 34). This threshold was 0.1 mA and 1 Hz and did not appear to vary significantly between individual animals; this was consistent with the previous paradigm in rats (0.1–0.3 mA) (35, 36) We found that eye movement and facial muscle response were similar between individuals when monitored by camera. Therefore, we kept this threshold for all experimental mice. When computer-controlled sinusoidal stimulation was applied, the amplitude of current was cyclical and was varied between −0.1 mA and +0.1 mA (threshold) via a DC-shifted device (A-M Systems Model 2200 Analog Stimulus Isolator) in which the opto-electrical isolation allocated the cathode-anode, the output waveform followed input, and the amplitude of the subthreshold current was altered sinusoidally from 0 to −0.1 mA in the cathode (the right) and from 0 to +0.1 mA in the anode (the left). Therefore, we used a sinusoidal DC-shifted GVS current of 0.1 mA and 1 Hz for the intervention. The mice in the non-GVS group were also restrained by the same procedure as in the GVS group but without current. GVS was delivered to the mice in the GVS group for 5 days, with 30-min sessions each day from POD 0 within 5 h after UL to POD 4 in a head-restrained awake state (Figure 1).

### Open Field Tasks

**Figure 2:**
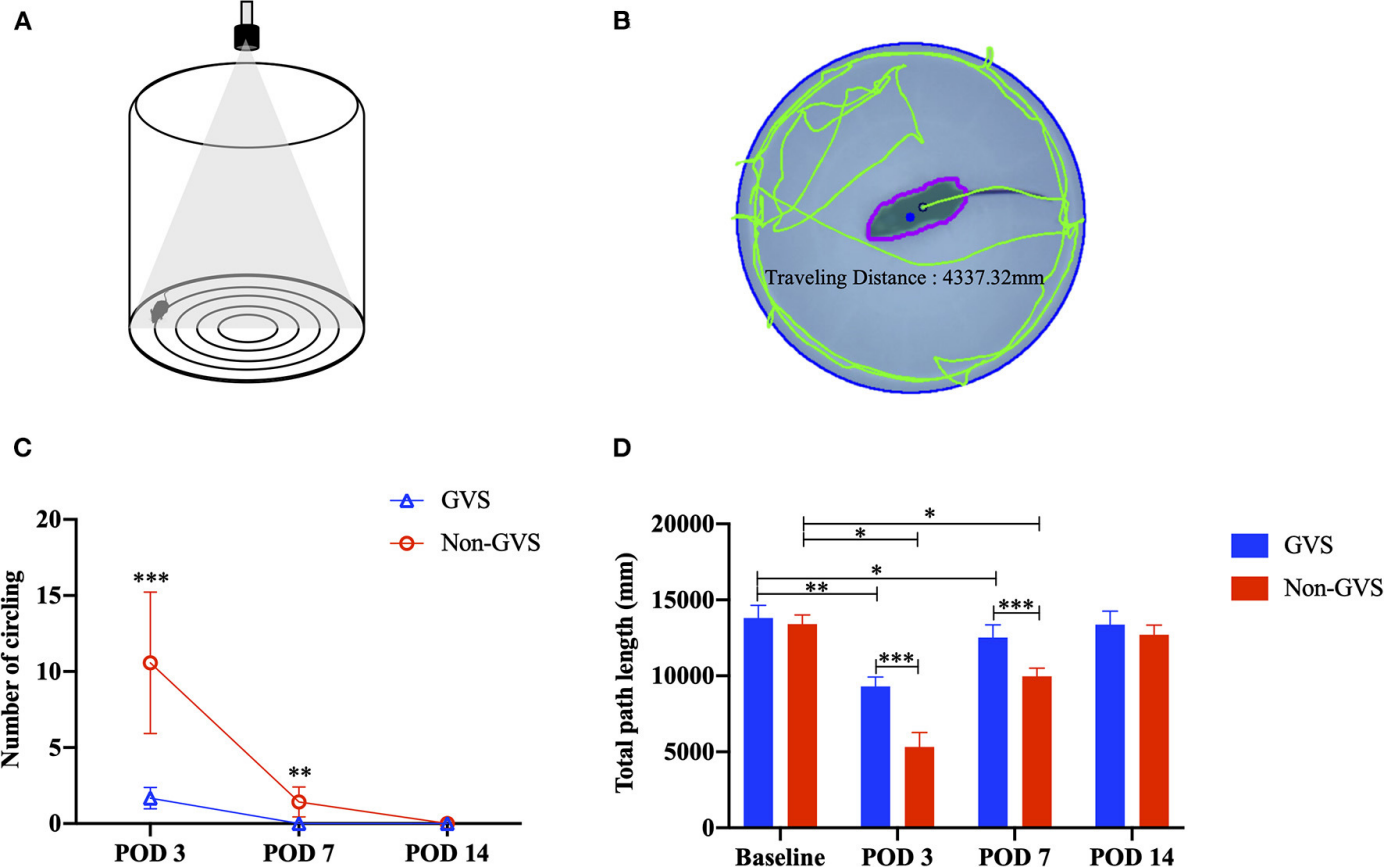
The open-field task comprising a circular arena of a white plastic cylinder 37 cm in diameter and 53 cm in height **(A)**. The recorded images were processed with a digital video-based tracking system using an image subtraction technique: the green lines indicate the total path length **(B)**. Evaluation of the behavioral changes of static vestibular function assessed by intermittent circling **(C)** and dynamic vestibular function reflected by the total path length **(D)** through an open field task. In the non-GVS group, intermittent circling in place was exhibited until POD 7 **(C)** and the total path length was also significantly deceased until POD 7 compared to the baseline value **(D)**. However, GVS improved the amount of circling and the total path length during the acute phase of UL. GVS, galvanic vestibular stimulation; POD, postoperative day; UL, unilateral labyrinthectomy. *p < 0.05; **p < 0.01; ***p < 0.001.

Mice were tested for 2 min in an open field apparatus comprising a circular arena of a white plastic cylinder 37 cm in diameter and 53 cm in height, which was illuminated with red light from the top at the center of the apparatus (Figure 2A) (37). To start each test trial, the mice were individually introduced to the center and tracked by an overhead camera HD 1080p C920 (Logitech, Switzerland) with a sampling rate of 30 frames per second. The locomotor activities of the mice were assessed according to the amount of circling (stereotyped rotatory movement in circles around the animal's hips) and total path length traveled across the whole device ground (mm). The ground was divided into the inner (central) and outer (peripheral) zones, and the percentage of time spent in the outer zone was used as an indicator for anxiety. The recorded images were processed with a customized analysis package (Figure 2B) (37).

### Statistical Analysis

All data were analyzed using SPSS Statistics version 23.0 (IBM Corp., Armonk, NY, USA). Values of significant difference were calculated using the Independent *t*-test or Mann–Whitney *U*-test for two group comparisons (GVS vs. non-GVS group) and paired *t*-test or Wilcoxon signed rank test for pairwise comparisons (baseline *vs*. PODs 3,7,14 values in each group) according to data distribution. The normal distribution of the data was verified by the Kolmogorov–Smirnov method. All the tests were performed at a 0.05 level of significance. Data were expressed as mean ± SD.

## Results

The baseline VOR data to sinusoidal stimulation before UL are presented in Table 1. There were no significant differences in gain, phase (degree), or symmetry (%) between the GVS and non-GVS groups. The number of times a mouse circled in place could not be calculated at baseline in either group because circling did not occur. The baseline locomotor activities assessed by total path length (mm) were similar between the two groups (Figures 2C,D).

**Table 1:** The VOR response to sinusoidal stimulation before UL in both GVS and non-GVS groups (the baseline).

	**Group**	**0.08 Hz**	**0.1 Hz**	**0.16 Hz**	**0.32 Hz**	**0.64 Hz**	**1 Hz**	**Total**
Gain	GVS group	0.80 ± 0.04	0.82 ± 0.03	0.85 ± 0.06	0.86 ± 0.05	0.85 ± 0.04	0.82 ± 0.05	0.83 ± 0.03
	Non-GVS group	0.74 ± 0.08	0.79 ± 0.05	0.86 ± 0.05	0.87 ± 0.05	0.88 ± 0.07	0.82 ± 0.05	0.83 ± 0.05
	*p*-value^a^	0.070	0.185	0.791	0.687	0.174	0.836	0.714
Phase	GVS group	21.90 ± 6.95	14.42 ± 4.82	10.79 ± 3.27	8.03 ± 2.15	7.30 ± 3.07	2.83 ± 2.22	10.88 ± 2.96
	Non-GVS group	24.65 ± 2.51	17.94 ± 2.99	10.80 ± 3.28	7.14 ± 3.53	5.55 ± 2.14	6.49 ± 4.27	12.10 ± 1.50
	*p*-value^a^	0.296	0.113	0.998	0.540	0.299	0.071	0.339
Symmetry	GVS group	1.80 ± 1.77	0.68 ± 0.38	0.78 ± 0.78	0.50 ± 0.26	0.41 ± 0.33	0.55 ± 0.41	0.79 ± 0.53
	Non-GVS group	0.43 ± 0.21	0.56 ± 0.24	0.67 ± 0.23	0.49 ± 0.34	0.47 ± 0.28	0.51 ± 0.24	0.52 ± 0.12
	*p*-value^a^	0.071	0.408	0.351	0.918	0.717	0.806	0.536

a *The average value of each GVS and non-GVS group was compared by an independent t-test or Mann–Whitney U-test according to data normality*.

*VOR, vestibulo-ocular reflex; UL, unilateral labyrinthectomy; GV, galvanic vestibular stimulation*.

### Temporal Changes of Locomotion and VOR in UL Mice

In the acute phase after UL, signs of UVD, such as spontaneous horizontal nystagmus beating toward the contralesional side, head-tilting, falling toward the ipsilesional side, backward gait, and clockwise circling, were observed. It took about 2 days after UL for the mice to regain a stable posture and walk steadily. Considering this natural recovery course, we conducted subsequent VOR investigations from POD 3, free from the limitations of motor coordination problems (Figure 1). After UVD, circling in place was exhibited until POD 7 (Figure 2C, non-GVS group), and the total path length was significantly decreased at POD 3 (from 13406.5 ± 609.8 mm at baseline to 5323.16 ± 953.42 mm, *p* < 0.05, Mann–Whitney *U*-test) and POD 7, and then gradually returned to the baseline level at POD 14 (*p* = 0.128, Wilcoxon signed rank test) (Figure 2D) during open field tasks.

**Figure 3:**
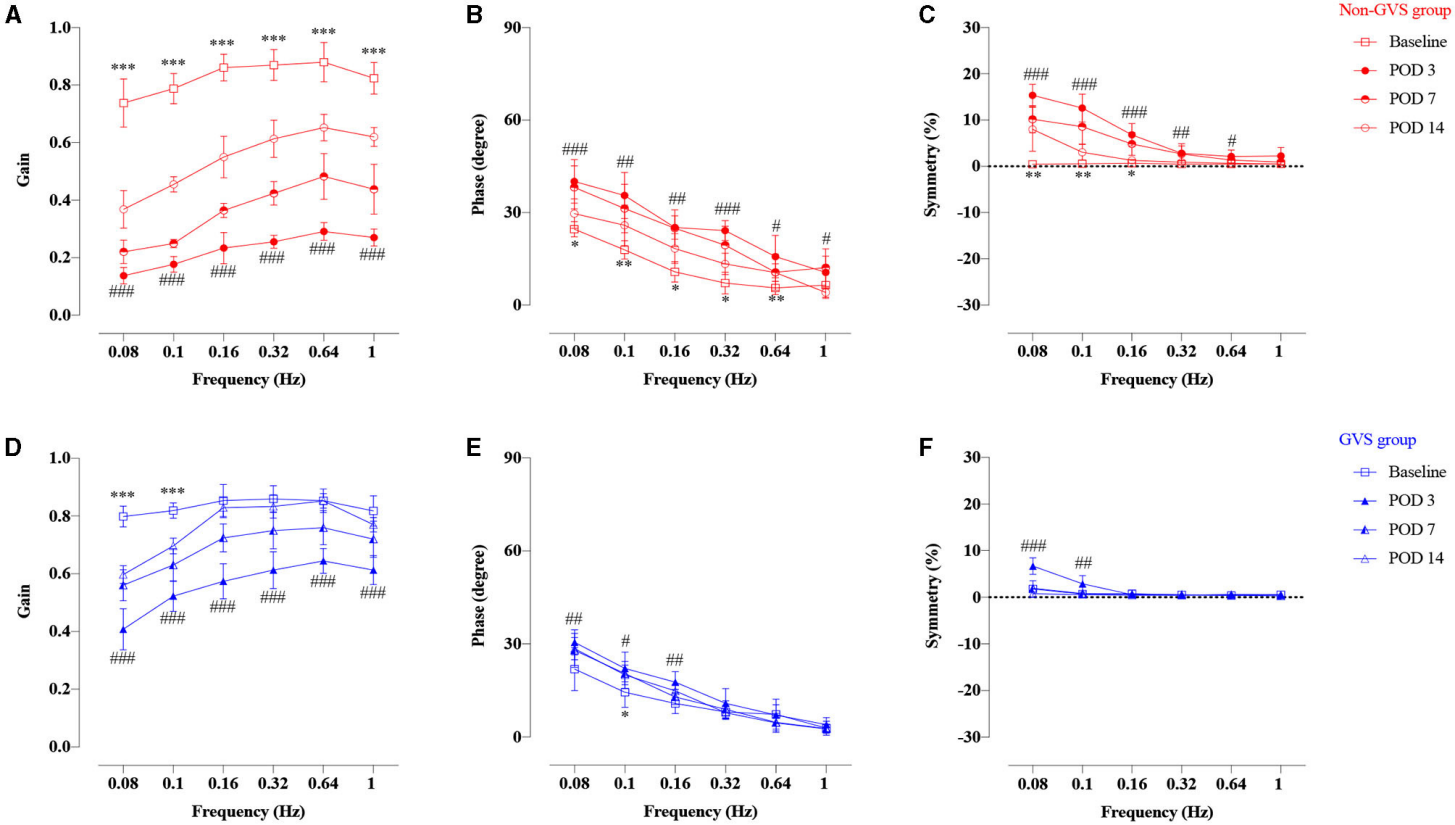
Time course of the compensation process of VOR responses of gain, phase, and symmetry of the non-GVS group (**A–C** with red colors) and the GVS group (**D–F** with blue colors) compared to the baseline value. Marked VOR gain reduction was observed at all frequencies at POD 3 in the non-GVS group (###*p* < 0.001, paired *t*-test). Although the VOR gain increased considerably at POD 14, it still remained impaired at all frequencies compared to baseline value (***p < 0.001, paired *t*-test) **(A)**. The phase and symmetry also gradually recovered over time up to POD 14, but they did not reach the values of the baseline **(B,C)**. The GVS group also exhibited VOR gain reduction at all frequencies at POD 3 after applying three sessions of GVS compared to the baseline (###*p* < 0.0001, paired *t*-test) **(D)**. However, the VOR gain was gradually recovered until POD 14 and nearly reached the value of the baseline, especially at high-frequency stimuli **(D)**. The phase and asymmetry of VOR were fully compensated at POD 14, with the exception of the phase at 0.1 Hz (*p = 0.023, Wilcoxon signed rank test) **(E,F)**. VOR, vestibulo-ocular reflex; GVS, galvanic vestibular stimulation; UL, unilateral labyrinthectomy; POD, postoperative day. *A significant difference between the values of the baseline and the values at POD 14; #A significant difference between the values of the baseline and the values at POD 3. *, # indicate *p* < 0.05; **, ## indicate *p* < 0.01; ***, ### indicate *p* < 0.001.

The VOR responses for gain, phase (degree), and symmetry (%) to sinusoidal vestibular stimulation before (baseline) and after UVD are shown in Figure 3 (non-GVS group, red color). At the baseline before surgery, the mean VOR gain increased with frequency, from 0.74 ± 0.08 at 0.08 Hz to 0.82 ± 0.05 at 1 Hz. The phase of VOR showed a phase lead at lower frequencies of stimulation that normalized as the frequency increased, from 24.6 ± 2.5 at 0.08 Hz to 6.5 ± 4.3 at 1 Hz. Asymmetry was minimally observed, at an average of 0.52%, to the right side over the entire frequency before UL (Table 1, Figure 3, red squares).

Temporal changes of VOR responses during sinusoidal rotations reflect the natural courses of vestibular restoration after UVD (Figure 3, non-GVS group). The VOR gain dropped significantly at all tested frequencies immediately after UL. VOR gains were reduced at POD 3 by an average of 72% compared to the baseline value (*p* < 0.001, paired *t*-test, 0.14 ± 0.03 at 0.08 Hz and 0.29 ± 0.03 at 0.64 Hz) and at POD 7 by 57% compared to baseline (*p* < 0.001, paired *t*-test) (Figure 3A). Although the VOR gain increased considerably at POD 14 to a maximum of 0.65 ± 0.05 at 0.64 Hz, it still remained impaired at all frequencies compared to the baseline value (*p* < 0.001, paired *t*-test) (Figure 3A, red open circles). The phase and symmetry also gradually recovered over time up to POD 14, but they did not reach the values of the baseline (Figures 3B,C). The phase lead at POD 14 was significantly different from the values of the baseline value at most frequencies except 1 Hz (*p* = 0.245, paired *t*-test) (Figure 3B). Asymmetry was apparent at low frequencies from 0.08 to 0.16 Hz, but became symmetric at 0.32–1 Hz during the examination days (Figure 3C). Overall, the symmetry of VOR improved within 2 weeks after UL, especially at high frequencies, but the values of VOR gain and phase remained significantly impaired compared to the baseline value (Figures 3A–C).

### GVS Effects on Locomotion and VOR in UL Mice

Changes in locomotor activities after GVS intervention (the GVS group) were significant during the acute phase after UL. In the GVS group, intermittent circling behavior at POD 3 was observed only 1.67 times, and no abnormal behaviors were observed at POD 7 or 14 (Figure 2C). The total path length also decreased from 13803.1 ± 840.9 4 mm to 9309.4 ± 607.6 mm at POD 3 (*p* = 0.008), and recovered to the baseline value at POD 14 (*p* = 0.441, Wilcoxon signed rank test) (Figure 2D).

**Figure 4:**
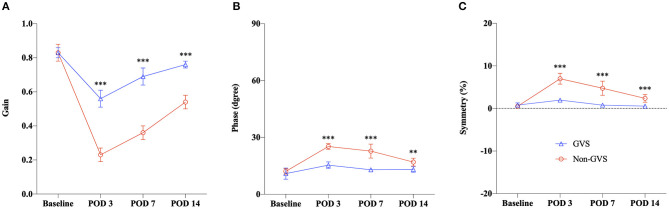
Summary of the VOR recovery for the GVS vs. the non-GVS group. The VOR responses were compared by averaging the values across frequencies. We observed marked differences in improvement across the time course between the UL with (the GVS group) and without GVS intervention (the non-GVS group), as indicated by Gain **(A)**, Phase **(B)**, and Symmetry **(C)**. The recovery of VOR responses in the GVS group was significantly faster than the non-GVS group from POD 3, and this trend was maintained until POD 14. VOR, vestibulo-ocular reflex; GVS, galvanic vestibular stimulation; UL, unilateral labyrinthectomy; POD, postoperative day. **p < 0.01; ***p < 0.001.

After GVS intervention, temporal changes of VOR responses are shown in Figure 3 (GVS group, blue color). At POD 3, after the UL mice had received three sessions of GVS in the GVS group, VOR gain reduction was significantly less attenuated compared to the non-GVS group (Figures 3D, 4A). The VOR gain gradually recovered during the follow-up days and nearly reached the value of the baseline, especially at high-frequency stimuli (0.16–1 Hz), at POD 14 (Figure 3D). After GVS intervention, the phase of VOR showed similar values with the baseline at PODs 7 (Figure 3E, Supplementary Table 3, *p* = 0.074, paired *t*-test) and 14 (Figure 3E, Supplementary Table 4, *p* = 0.063, paired *t*-test). The phase lead of the GVS group was significantly decreased compared to the non-GVS group across all frequencies from POD 3 (Figure 4B, Supplementary Table 2). The phase lead of the non-GVS group was also gradually compensated, but still observed at some frequencies at POD 14 compared to the GVS group (at 0.32 Hz, *p* = 0.002 and at 0.64 Hz, *p* = 0.001, Mann–Whitney *U*-test, Figure 4B, Supplementary Table 4). Similar to the phase response, after GVS intervention, the asymmetry pattern was recovered to the level of the baseline value at POD 3, which was asymmetric only at low frequencies of 0.08 Hz (*p* = 0.004, paired *t*-test) and 0.1 Hz (*p* = 0.012, Wilcoxon signed rank test), and was fully compensated at POD 14 (Figure 3F).

A comparison of the time course of VOR responses between the UL mice with (the GVS group) and without GVS intervention (the non-GVS group) revealed positive effects of sinusoidal GVS for improving the VOR parameters in UL mice (Figure 4). From POD 3, when the UL mice had received three sessions of GVS, the GVS group showed marked improvement in the VOR gain compared to the non-GVS group at all frequencies (Figure 4A, Supplementary Table 2). Gain reduction in the GVS group was significantly less attenuated from POD 3, after just 3 sessions of GVS, to POD 14, after the completion of five sessions of GVS, compared to the non-GVS group; gain reduction was about 34% of baseline in the GVS group but 73% in the non-GVS group at POD 3 (Figure 4A). Although VOR gain was recovered substantially after UVD in both GVS and non-GVS groups, GVS intervention demonstrated a significant effect on gain recovery compared to the non-GVS group during the examined days. Meanwhile, the phase and symmetry of VOR also showed GVS effects, where the values of the GVS group compensated those seen at baseline, while those of the non-GVS group remained impaired significantly (Figures 3B,C,E,F). At POD 14, the GVS group had almost recovered gain, phase and symmetry of the VOR to the baseline values; however, the non-GVS group remained significantly less compensated compared to the baseline values and the GVS group (Figures 3, 4).

## Discussion

The current study demonstrates the clear effects of GVS intervention on locomotion and VOR recoveries induced by UVD in the mouse model.

### The Effects of GVS Intervention on the Recovery of Static and Dynamic Deficits

Dynamic deficits, such as VOR asymmetry, postural instability, and disequilibrium, are incompletely and slowly compensated compared to static imbalance (38, 39). The dynamic deficits are less dependent on the rebalancing of VN on both sides and require newly elaborated sensorimotor reorganizations at multisensory brain regions (38, 40). In case the dynamic deficits are not properly compensated, impaired balance control and oscillopsia persist due to decreased VOR gain and phase shift. A previous study showing the positive effects of GVS on the static and dynamic vestibular compensation in rats after UL suggested a mechanism of neurogenesis via compensation for the loss of irregular phasic signal and accelerated vestibular adaption (10). In a study using a hemi-parkinsonian rat model, noisy-GVS intervention improved the dynamic deficits due to facilitatory effects on the vestibulo-spinal pathway and other dopamine-independent pathways, possibly by increasing GABA concentrations in ipsilesional substantia nigra, which was attributed to the neuromodulation mechanism (41). An additional study showed that the recovery process for static and dynamic deficits induced by a UVD model takes at least 36 days (42). Taken together, our current study showing significant improvements in locomotion, reflected by circling behavior and the total path length after GVS intervention, demonstrates the positive effects of GVS application on dynamic postural control during the acute phase after UVD.

### The Recovery of VOR in GVS and Non-GVS Mice

Notably, the parameters of VOR gain, phase, and symmetry in the GVS group nearly returned to the baseline values by the second week, whereas the values in the non-GVS group recovered slowly but remained significantly impaired compared to the values of the GVS group and the baseline. This effect of GVS on the recovery of VOR is initiated from an acute stage of 3 days after UL, by which point the mice had undergone three sessions of GVS. This effect was maintained until the 14th day, when the final recovery of VOR gain was up to 92% of the baseline value in the GVS group compared to 65% in the non-GVS group. Along with improvement of locomotor activities assessed by intermittent circling behavior and the total path length after GVS intervention at the acute phase of UL, this finding suggests that GVS application was effective in the recovery of static and dynamic vestibular deficits via modulating the VOR pathways, starting at the early stages after UVD. The current finding of a natural recovery after UL without GVS intervention is consistent with previous studies that showed a VOR gain reduction by nearly 75% on the first day that recovered to about 80% on the 10th day after UL, but never fully recovered, in a non-GVS group (30, 43, 44). However, the different levels of VOR recovery among these studies suggest that recovery has considerable inter-individual variability in rodent species depending on the amount of vestibular deafferentation (30).

### Possible Mechanisms Behind GVS Restoration of Imbalanced Vestibular Nuclei

While GVS has been considered to directly activate primary vestibular afferents at the spike trigger zone that is located between the vestibular sensory epithelium and the afferent terminals (1, 15), recent evidence suggests that GVS-induced vestibular responses also recruit vestibular hair cell activation (8, 11). Thus, to account for various effects evoked by GVS, the mechanisms acting on the synapse itself as well as post-synaptic mechanisms should be considered. UVD induces a strong imbalance in the resting discharges of the vestibular nuclei (VN) complex on each side, with different reactive mechanisms taking place simultaneously at both the central and peripheral vestibular structures (38, 45, 46). Unilateral peripheral vestibular deafferentation causes a drop of the spontaneous firing rate and sensitivity of the type I VN neurons in the ipsilesional medial VN, and an increased inhibitory drive from the contralesional side through the inhibitory commissural pathways, which enhances the imbalance. The key point of vestibular compensation is to restore the damaged activity of the ipsilateral VN and the balanced activity between the two sides (38). Recent investigations aimed at visualizing the relative changes of glucose metabolism (rCGM) showed a significant asymmetry in the VN complexes and related structures of the vestibulo-cerebellum, thalamus, vestibular cortex, hippocampus, and amygdala in the acute stage of UVD, followed by re-balanced rCGM in these structures (47). In rodents, signs of unilateral vestibular loss, such as spontaneous horizontal nystagmus beating toward the contralesional side, head-tilting, falling toward the ipsilesional side, backward gait, and clockwise circling, are generally fully compensated within 1 or 2 weeks (30, 48). These static deficits are explained by the asymmetrical resting discharge between both VN, and their recovery is achieved by returning their symmetrical firing rates on both sides. It was found that cathodal and anodal GVS predominantly activate and reduce the thick fast-conducting irregularly firing afferents, respectively (4–9), and these fibers are preferentially connected with the vestibulo-spinal neurons (6) that are responsible for the postural asymmetries after UVD (48–50). In the current study, the static deficit assessed by intermittent circling behavior showed better results in the GVS intervention group at the acute stage of UL, suggesting that GVS can restore postural asymmetry via modulating type I hair cells with an irregular phasic signal (1, 5, 7). Another possible explanation for the static compensation after UL is that a controlled GVS stimulation with the right cathode stimulation for excitation and the left anode stimulation for inhibition in the vestibular afferents could constructively modulate the vestibular commissural inhibitory system and might have a positive effect on central adaptation for facilitating the vestibular compensation (38).

### Properties of GVS-Induced Responses

The ameliorating effects of sinusoidal GVS on the VOR observed here should take into account the fact that GVS directly modulates neurotransmitter release by hair cells as well as the vestibular nucleus (8, 11). Studies showing the loss of galvanically induced VOR in patients with intractable Meniere's disease and systemic gentamicin vestibulopathy provide further indications that GVS can activate hair cells in addition to vestibular afferents (51, 52). Definitive evidence for GVS activation of vestibular hair cells came from a pharmacological study on a *Xenopus laevis* tadpole model using a bath application of glutamatergic antagonists. During a block of glutamatergic transmission for a controlled block of the synaptic transmission between hair cells and vestibular afferents, they showed that GVS induced discharge modulation (8). They also showed that the lowest GVS current seems to be more effective to recruit hair cells, whereas larger currents are effective in modulating the activity of afferents, indicating the efficacy of sub-threshold GVS to the non-linear system of the peripheral vestibular system as a stochastic resonance (53). The cellular mechanisms underlying the beneficial properties of GVS were determined using a computational model of the vestibular end organ that elicited all experimentally observed response characteristics to GVS simultaneously (54). As a result, GVS was shown to affect both hair cell vesicle release and axonal excitability simultaneously to maintain natural firing statistics (54). This process revealed that GVS may have certain advantages in prosthetic replication compared with pulsatile stimulation (artificial activation of the axons): *(i)* GVS has an effect on axons as well as end organs and smaller receptor cells, which are associated with vestibular inputs further upstream in neural processing, allowing for potentially more natural responses that engage the same molecular and cellular machinery as the normally functioning physiological system; *(ii)* GVS can induce graded amounts of excitation or inhibition through membrane potential changes that can match the natural system firing rates; and *(iii)* GVS can capture natural stochastic firing patterns that could be important to the system (54). Among the various electrical currents for GVS, such as current steps (5), sinusoids (8, 9), or band-limited noise (18, 24), the sinusoidal currents applied in this study are more suitable to stimulate a natural head motion and represent a more dynamic stimulus condition compared with current steps (1, 8, 9). Although little is known as to how such an electrical waveform modifies the neuronal activity of vestibular hair cells and/or afferent fibers, the timing of the responses to sinusoidal GVS corresponds with velocity, showing similar phase relations between GVS and head-motion evoked responses in VOR neuronal elements (8). The sinusoidal GVS has properties of phase advanced responses in which the phase lead in low frequency becomes lagged as it goes to high frequency, which might be related to neuronal integration processes along with the VOR pathway (8). However, the question of which particular subthreshold GVS current is the most beneficial should be addressed in future research. A previous study, which quantified the frequency and amplitude response of postural sway to sinusoidal GVS, indicated that there was an increase of postural response when the stimulus increased from 0.05 to 0.5 mA, but was unchanged from 0.5 to 1 mA. The saturation effect at 0.5 mA was proposed as an explanation in that case (55). Meanwhile, another experiment, which was conducted to compare the efficacy of high- and low-frequency GVS, found that the impact of high-rate GVS on behavioral recovery was not different from that of low-rate GVS. However, cell proliferation was significantly higher in the high-rate GVS group (10). High-frequency GVS preferentially activates otolithic afferents and the reticulo-spinal pathway, which is a stronger input for adjusting standing posture, while low-frequency GVS preferentially activates semicircular canal afferents and the vestibulo-spinal pathway (56).

We developed this GVS intervention paradigm as a daily 30-min session for 5 days based on evidence from previous studies. One GVS trial in rats with bilateral vestibular lesions used an intervention model of 30 min/session/day, and effects on spatial cognition were observed in the five-session (5-day) group but not in the single-session (1-day) group (36). Another study, which also used the protocol of 30 min/day for 14 days, showed a GVS effect accelerating static and dynamic vestibular compensation, as well as increasing vestibular nuclei cell proliferation (10). In the current investigation, in order to determine the efficacy of GVS in the acute phase post-UL as well as its lasting effects, GVS current was initiated 5 h post-UL and sustained for a 30-min session daily for 5 days combined with a 2-week follow-up period. To minimize the adverse effects of GVS intervention, i.e., nausea, mild vertigo, discomfort, eyestrain, headache, head fullness, and blurred vision, (57) it is preferable to use the minimum number of sessions necessary to achieve optimal therapeutic efficacy. Indeed, a previous study reported that a small number of GVS sessions was sufficient to induce lasting changes in tactile extinction that remained stable for at least 2.8 months (58) or even 1 year post-stimulation (59). Of course, further evaluation of intervention time should be included in our future research, which will examine the efficacy of GVS in models of subacute or chronic vestibular injuries with a longer follow-up duration.

In conclusion, this study shows that GVS intervention accelerates static and dynamic vestibular compensation after UVD by improving VOR and motor coordination during the acute phase in a UVD mouse model. The current findings have important clinical implications suggesting that early intervention with GVS may be effective for the management of impaired postural control and gaze stability in patients with acute unilateral vestibular damage.

## Data Availability Statement

The original contributions generated for the study are included in the article/Supplementary Material, further inquiries can be directed to the corresponding author/s.

## Ethics Statement

The animal procedures included in this study were consistent with the Assessment and Accreditation of Laboratory Animal Care International and have been reviewed and approved by the Animal Care Committee of the Gachon University of Medicine and Science (IRB MRI 2019-0008).

## Author Contributions

S-YO and GCH conceived and planned the experiments. G-SN and TTN carried out the experiments. TTN and J-JK planned and carried out the simulations. S-YO, G-SN, and GCH contributed to the interpretation of the results. G-SN and TTN took the lead in writing the manuscript. All authors provided critical feedback and helped shape the research, analysis and manuscript.

## Conflict of Interest

The authors declare that the research was conducted in the absence of any commercial or financial relationships that could be construed as a potential conflict of interest.

## Publisher's Note

All claims expressed in this article are solely those of the authors and do not necessarily represent those of their affiliated organizations, or those of the publisher, the editors and the reviewers. Any product that may be evaluated in this article, or claim that may be made by its manufacturer, is not guaranteed or endorsed by the publisher.

## References

[1] Goldberg JM Smith CE Fernández C. Relation between discharge regularity and responses to externally applied galvanic currents in vestibular nerve afferents of the squirrel monkey. J Neurophysiol. (1984) 51:1236–56. 10.1152/jn.1984.51.6.1236 6737029

[2] Courjon JH Precht W Sirkin DW. Vestibular nerve and nuclei unit responses and eye movement responses to repetitive galvanic stimulation of the labyrinth in the rat. Exp Brain Res. (1987) 66:41–8. 10.1007/BF00236200 3582534

[3] Utz KS Dimova V Oppenländer K Kerkhoff G. Electrified minds: transcranial direct current stimulation (tDCS) and galvanic vestibular stimulation (GVS) as methods of non-invasive brain stimulation in neuropsychology–a review of current data and future implications. Neuropsychologia. (2010) 48:2789–810. 10.1016/j.neuropsychologia.2010.06.002 20542047

[4] Curthoys IS Macdougall HG. What galvanic vestibular stimulation actually activates. Front Neurol. (2012) 3:117. 10.3389/fneur.2012.00117 22833733 PMC3400934

[5] Kim J Curthoys IS. Responses of primary vestibular neurons to galvanic vestibular stimulation (GVS) in the anaesthetised guinea pig. Brain Res Bull. (2004) 64:265–71. 10.1016/j.brainresbull.2004.07.008 15464864

[6] Dlugaiczyk J Gensberger KD Straka H. Galvanic vestibular stimulation: from basic concepts to clinical applications. J Neurophysiol. (2019) 121:2237–55. 10.1152/jn.00035.2019 30995162

[7] Kwan A Forbes PA Mitchell DE Blouin JS Cullen KE. Neural substrates, dynamics and thresholds of galvanic vestibular stimulation in the behaving primate. Nat Commun. (2019) 10:1904. 10.1038/s41467-019-09738-1 31015434 PMC6478681

[8] Gensberger KD Kaufmann AK Dietrich H Branoner F Banchi R Chagnaud BP. Galvanic vestibular stimulation: cellular substrates and response patterns of neurons in the vestibulo-ocular network. J Neurosci. (2016) 36:9097–10. 10.1523/JNEUROSCI.4239-15.2016 27581452 PMC6601907

[9] Kim KS Minor LB Della Santina CC Lasker DM. Variation in response dynamics of regular and irregular vestibular-nerve afferents during sinusoidal head rotations and currents in the chinchilla. Exp Brain Res. (2011) 210:643–9. 10.1007/s00221-011-2600-8 21369854 PMC4010622

[10] Shaabani M Lotfi Y Karimian SM Rahgozar M Hooshmandi M. Short-term galvanic vestibular stimulation promotes functional recovery and neurogenesis in unilaterally labyrinthectomized rats. Brain Res. (2016) 1648:152–62. 10.1016/j.brainres.2016.07.029 27444558

[11] Norris CH Miller AJ Perin P Holt JC Guth PS. Mechanisms and effects of transepithelial polarization in the isolated semicircular canal. Hear Res. (1998) 123:31–40. 10.1016/S0378-5955(98)00096-3 9745953

[12] Holstein GR Friedrich VL Jr Martinelli GP Ogorodnikov D Yakushin SB. Fos expression in neurons of the rat vestibulo-autonomic pathway activated by sinusoidal galvanic vestibular stimulation. Front Neurol. (2012) 3:4. 10.3389/fneur.2012.00004 22403566 PMC3289126

[13] Samoudi G Nilsson A Carlsson T Bergquist F. c-Fos expression after stochastic vestibular stimulation and levodopa in 6-OHDA hemilesioned rats. Neuroscience. (2020) 424:146–54. 10.1016/j.neuroscience.2019.10.039 31704349

[14] Dilda V MacDougall HG Curthoys IS Moore ST. Effects of Galvanic vestibular stimulation on cognitive function. Exp Brain Res. (2012) 216:275–85. 10.1007/s00221-011-2929-z 22076407

[15] Fitzpatrick RC Day BL. Probing the human vestibular system with galvanic stimulation. J Appl Physiol. (2004) 96:2301–16. 10.1152/japplphysiol.00008.2004 15133017

[16] Yamamoto Y Struzik ZR Soma R Ohashi K Kwak S. Noisy vestibular stimulation improves autonomic and motor responsiveness in central neurodegenerative disorders. Ann Neurol. (2005) 58:175–81. 10.1002/ana.20574 16049932

[17] Pal S Rosengren SM Colebatch JG. Stochastic galvanic vestibular stimulation produces a small reduction in sway in Parkinson's disease. J Vestib Res. (2009) 19:137–42. 10.3233/VES-2009-0360 20448339

[18] Wuehr M Nusser E Decker J Krafczyk S Straube A Brandt T. Noisy vestibular stimulation improves dynamic walking stability in bilateral vestibulopathy. Neurology. (2016) 86:2196–202. 10.1212/WNL.0000000000002748 27164706

[19] Kim HJ Choi JY Son EJ Lee WS. Response to galvanic vestibular stimulation in patients with unilateral vestibular loss. Laryngoscope. (2006) 116:62–6. 10.1097/01.mlg.0000184525.14825.f4 16481811

[20] Aw ST Todd MJ Lehnen N Aw GE Weber KP Eggert T. Electrical vestibular stimulation after vestibular deafferentation and in vestibular schwannoma. PLoS One. (2013) 8:e82078. 10.1371/journal.pone.0082078 24349188 PMC3861342

[21] Pavlik AE Inglis JT Lauk M Oddsson L Collins JJ. The effects of stochastic galvanic vestibular stimulation on human postural sway. Exp Brain Res. (1999) 124:273–80. 10.1007/s002210050623 9989432

[22] Mulavara AP Fiedler MJ Kofman IS Wood SJ Serrador JM Peters B. Improving balance function using vestibular stochastic resonance: optimizing stimulus characteristics. Exp Brain Res. (2011) 210:303–12. 10.1007/s00221-011-2633-z 21442221

[23] Wuehr M Decker J Schniepp R. Noisy galvanic vestibular stimulation: an emerging treatment option for bilateral vestibulopathy. J Neurol. (2017) 264:81–6. 10.1007/s00415-017-8481-4 28391373

[24] Iwasaki S Yamamoto Y Togo F Kinoshita M Yoshifuji Y Fujimoto C. Noisy vestibular stimulation improves body balance in bilateral vestibulopathy. Neurology. (2014) 82:969–75. 10.1212/WNL.0000000000000215 24532279

[25] Iwasaki S Karino S Kamogashira T Togo F Fujimoto C Yamamoto Y. Effect of noisy galvanic vestibular stimulation on ocular vestibular-evoked myogenic potentials to bone-conducted vibration. Front Neurol. (2017) 8:26. 10.3389/fneur.2017.00026 28217106 PMC5290309

[26] Péricat D Farina A Agavnian-Couquiaud E Chabbert C Tighilet B. Complete and irreversible unilateral vestibular loss: a novel rat model of vestibular pathology. J Neurosci Methods. (2017) 283:83–91. 10.1016/j.jneumeth.2017.04.001 28390798

[27] Chang MY Park MK Park SH Suh MW Lee JH Oh SH. Surgical labyrinthectomy of the rat to study the vestibular system. J Vis Exp. (2018) 57681. 10.3791/57681 29863682 PMC6101262

[28] Kim MS Kim JH Jin YZ Kry D Park BR. Temporal changes of cFos-like protein expression in medial vestibular nuclei following arsanilate-induced unilateral labyrinthectomy in rats. Neurosci Lett. (2002) 319:9–12. 10.1016/S0304-3940(01)02422-3 11814641

[29] Kim MJ Kim N Lee EJ Han GC. “Tail-Hanging Test” behavioral parameter of vestibular deficit and compensation in labyrinthectomized mouse model. J Int Adv Otol. (2012) 8:453.

[30] Beraneck M McKee JL Aleisa M Cullen KE. Asymmetric recovery in cerebellar-deficient mice following unilateral labyrinthectomy. J Neurophysiol. (2008) 100:945–58. 10.1152/jn.90319.2008 18509072 PMC2525728

[31] Simon F Pericat D Djian C Fricker D Denoyelle F Beraneck M. Surgical techniques and functional evaluation for vestibular lesions in the mouse: unilateral labyrinthectomy (UL) and unilateral vestibular neurectomy (UVN). J Neurol. (2020) 267:51–61. 10.1007/s00415-020-09960-8 32556569 PMC7718198

[32] Kim MJ Lee J Hong EJ Lee EJ Min YJ Lee DJ. Vestibulo-ocular reflex recordings of small rodents using a novel marker array. Res Vestibular Sci. (2016) 15:11–6.

[33] Minor LB Goldberg JM. Vestibular-nerve inputs to the vestibulo-ocular reflex: a functional-ablation study in the squirrel monkey. J Neurosci. (1991) 11:1636–48. 10.1523/JNEUROSCI.11-06-01636.1991 2045879 PMC6575423

[34] Zheng Y Geddes L Sato G Stiles L Darlington CL Smith PF. Galvanic vestibular stimulation impairs cell proliferation and neurogenesis in the rat hippocampus but not spatial memory. Hippocampus. (2014) 24:541–52. 10.1002/hipo.22247 24449222

[35] Best C Lange E Buchholz H-G Schreckenberger M Reuss S Dieterich M. Left hemispheric dominance of vestibular processing indicates lateralization of cortical functions in rats. Brain Struct Funct. (2014) 219:2141–58. 10.1007/s00429-013-0628-1 23979449

[36] Ghahraman MA Zahmatkesh M Pourbakht A Seifi B Jalaie S Adeli S. Noisy galvanic vestibular stimulation enhances spatial memory in cognitive impairment-induced by intracerebroventricular-streptozotocin administration. Physiol Behav. (2016) 157:217–24. 10.1016/j.physbeh.2016.02.021 26892259

[37] Kim MJ Hwang HJ Chung SW Han GC. Measuring the behavioral parameters of mouse following unilateral labyrinthectomy in round free field using an infrared lamp and a simple webcam camera. Res Vestibular Sci. (2011) 10:12–8.

[38] Lacour M Helmchen C Vidal PP. Vestibular compensation: the neuro-otologist's best friend. J Neurol. (2016) 263(Suppl. 1):S54–64. 10.1007/s00415-015-7903-4 27083885 PMC4833803

[39] Lacour M. Restoration of vestibular function: basic aspects and practical advances for rehabilitation. Curr Med Res Opin. (2006) 22:1651–9. 10.1185/030079906X115694 16968568

[40] Newlands SD Dara S Kaufman GD. Relationship of static and dynamic mechanisms in vestibuloocular reflex compensation. Laryngoscope. (2005) 115:191–204. 10.1097/01.mlg.0000154718.80594.2e 15689735

[41] Samoudi G Nissbrandt H Dutia MB Bergquist F. Noisy galvanic vestibular stimulation promotes GABA release in the substantia nigra and improves locomotion in hemiparkinsonian rats. PLoS ONE. (2012) 7:e29308. 10.1371/journal.pone.0029308 22238601 PMC3253081

[42] Liberge M Manrique C Bernard-Demanze L Lacour M. Changes in TNFα, NFκB and MnSOD protein in the vestibular nuclei after unilateral vestibular deafferentation. J Neuroinflammation. (2010) 7:91. 10.1186/1742-2094-7-91 21143912 PMC3004876

[43] Vibert N de Waele C Escudero M Vidal PP. The horizontal vestibulo-ocular reflex in the hemilabyrinthectomized guinea-pig. Exp Brain Res. (1993) 97:263–73. 10.1007/BF00228695 8150045

[44] Shinder ME Perachio AA Kaufman GD. VOR and Fos response during acute vestibular compensation in the Mongolian gerbil in darkness and in light. Brain Res. (2005) 1038:183–97. 10.1016/j.brainres.2005.01.043 15757634

[45] Dutia MB. Mechanisms of vestibular compensation: recent advances. Curr Opin Otolaryngol Head Neck Surg. (2010) 18:420–4. 10.1097/MOO.0b013e32833de71f 20693901

[46] Tighilet B Bordiga P Cassel R Chabbert C. Peripheral vestibular plasticity vs central compensation: evidence and questions. J Neurol. (2019) 266:27–32. 10.1007/s00415-019-09388-9 31134376

[47] Zwergal A Schlichtiger J Xiong G Beck R Günther L Schniepp R. Sequential [(18)F]FDG μPET whole-brain imaging of central vestibular compensation: a model of deafferentation-induced brain plasticity. Brain Struct Funct. (2016) 221:159–70. 10.1007/s00429-014-0899-1 25269833

[48] Smith PF Curthoys IS. Mechanisms of recovery following unilateral labyrinthectomy: a review. Brain Res Brain Res Rev. (1989) 14:155–80. 10.1016/0165-0173(89)90013-1 2665890

[49] Highstein SM Goldberg JM Moschovakis AK Fernández C. Inputs from regularly and irregularly discharging vestibular nerve afferents to secondary neurons in the vestibular nuclei of the squirrel monkey. II. correlation with output pathways of secondary neurons. J Neurophysiol. (1987) 58:719–38. 10.1152/jn.1987.58.4.719 2445938

[50] Lambert FM Malinvaud D Gratacap M Straka H Vidal PP. Restricted neural plasticity in vestibulospinal pathways after unilateral labyrinthectomy as the origin for scoliotic deformations. J Neurosci. (2013) 33:6845–56. 10.1523/JNEUROSCI.4842-12.2013 23595743 PMC6618882

[51] De Waele C Meguenni R Freyss G Zamith F Bellalimat N Vidal PP. Intratympanic gentamicin injections for Meniere disease: vestibular hair cell impairment and regeneration. Neurology. (2002) 59:1442–4. 10.1212/WNL.59.9.1442 12427902

[52] Aw ST Todd MJ Aw GE Weber KP Halmagyi GM. Gentamicin vestibulotoxicity impairs human electrically evoked vestibulo-ocular reflex. Neurology. (2008) 71:1776–82. 10.1212/01.wnl.0000335971.43443.d9 19029517

[53] Moss F Ward LM Sannita WG. Stochastic resonance and sensory information processing: a tutorial and review of application. Clin Neurophysiol. (2004) 115:267–81. 10.1016/j.clinph.2003.09.014 14744566

[54] Steinhardt CR Fridman GY. Direct current effects on afferent and hair cell to elicit natural firing patterns. Iscience. (2021) 24:102205. 10.1016/j.isci.2021.102205 33748701 PMC7967006

[55] Latt L Sparto P Furman J Redfern M. The steady-state postural response to continuous sinusoidal galvanic vestibular stimulation. Gait Posture. (2003) 18:64–72. 10.1016/S0966-6362(02)00195-9 14654209

[56] Dakin CJ Son GML Inglis JT Blouin JS. Frequency response of human vestibular reflexes characterized by stochastic stimuli. J Physiol. (2007) 583:1117–27. 10.1113/jphysiol.2007.133264 17640935 PMC2277188

[57] Lenggenhager B Lopez C Blanke O. Influence of galvanic vestibular stimulation on egocentric and object-based mental transformations. Exp Brain Res. (2008) 184:211–21. 10.1007/s00221-007-1095-9 17717649

[58] Schmidt L Utz KS Depper L Adams M Schaadt AK Reinhart S. Now you feel both: galvanic vestibular stimulation induces lasting improvements in the rehabilitation of chronic tactile extinction. Front Human Neurosci. (2013) 7:90. 10.3389/fnhum.2013.00090 23519604 PMC3602932

[59] Kerkhoff G Hildebrandt H Reinhart S Kardinal M Dimova V Utz KS. A long-lasting improvement of tactile extinction after galvanic vestibular stimulation: two Sham-stimulation controlled case studies. Neuropsychologia. (2011) 49:186–95. 10.1016/j.neuropsychologia.2010.11.014 21094654

