# Supplementary material for: Effects of Galvanic Vestibular Stimulation on Vestibular Compensation in Unilaterally Labyrinthectomized Mice
Source: Front Neurol. 2021 Sep 3;12:736849. doi: 10.3389/fneur.2021.736849 (PMC8446527; doi:10.3389/fneur.2021.736849)
Supplement: Supplementary file 1 [file Data_Sheet_1.pdf]

Supplementary Table S1. GVS threshold determination in the pilot experiment.

**Mouse**	**GVS day**	**GVS (with a frequency of 1 Hz) amplitude (mA)**															
		**0**	**0.01**	**0.02**	**0.03**	**0.04**	**0.05**	**0.06**	**0.07**	**0.08**	**0.09**	**0.1**	**0.11**	**0.12**	**0.13**	**0.14**	**0.15**
**1**	1	-	-	-	-	-	-	-	-	-	-	+	+	+	++	x	x
**2**	1	-	-	-	-	-	-	-	-	-	-	-	+	+	+	++	x
**3**	1	-	-	-	-	-	-	-	-	-	+	+	+	++	x	x	x
**4**	1	-	-	-	-	-	-	-	-	-	-	+	+	+	++	x	x
**5**	1	-	-	-	-	-	-	-	-	-	-	+	+	++	x	x	x
**1**	2	-	-	-	-	-	-	-	-	-	-	+	+	++	x	x	x
**2**	2	-	-	-	-	-	-	-	-	-	-	-	+	+	++	x	x
**3**	2	-	-	-	-	-	-	-	-	-	+	+	++	x	x	x	x
**4**	2	-	-	-	-	-	-	-	-	-	-	+	+	+	++	x	x
**5**	2	-	-	-	-	-	-	-	-	-	-	-	+	+	++	x	x
**1**	3	-	-	-	-	-	-	-	-	-	-	+	+	++	x	x	x
**2**	3	-	-	-	-	-	-	-	-	-	-	+	+	+	++	x	x
**3**	3	-	-	-	-	-	-	-	-	-	+	+	+	++	x	x	x
**4**	3	-	-	-	-	-	-	-	-	-	-	-	+	+	++	x	x
**5**	3	-	-	-	-	-	-	-	-	-	-	-	+	+	++	x	x
**1**	4	-	-	-	-	-	-	-	-	-	-	+	+	+	++	x	x
**2**	4	-	-	-	-	-	-	-	-	-	-	+	+	++	x	x	x
**3**	4	-	-	-	-	-	-	-	-	-	+	+	++	x	x	x	x
**4**	4	-	-	-	-	-	-	-	-	-	-	+	+	++	x	x	x
**5**	4	-	-	-	-	-	-	-	-	-	-	-	+	+	++	x	x
**1**	5	-	-	-	-	-	-	-	-	-	-	+	+	++	x	x	x
**2**	5	-	-	-	-	-	-	-	-	-	-	+	+	+	++	x	x
**3**	5	-	-	-	-	-	-	-	-	-	-	+	+	+	++	x	x
**4**	5	-	-	-	-	-	-	-	-	-	-	+	+	+	++	x	x
**5**	5	-	-	-	-	-	-	-	-	-	-	-	+	+	++	x	x

GVS threshold: **0.1012** ± 0.06658 mA, min: 0.09 mA, max: 0.11 mA

GVS: galvanic vestibular stimulation; -: no nystagmus; +: nystagmus; ++: strong nystagmus with facial muscle activities; x: no GVS application

Supplementary Table S2. The VOR response to sinusoidal stimulation 3 days post UL in the GVS and the non-GVS groups compared to baseline.

		0.08 Hz	0.1Hz	0.16 Hz	0.32 Hz	0.64 Hz	1 Hz	Total
Gain	GVS	0.41±0.07	0.52±0.05	0.57±0.06	0.61±0.06	0.64±0.04	0.61±0.05	0.56±0.05
	Non-GVS	0.14±0.03	0.18±0.03	0.23±0.05	0.26±0.02	0.29±0.03	0.27±0.03	0.23±0.03
	p-value[a]	<0.001	<0.001	<0.001	<0.001	<0.001	<0.001	<0.001
	p-value[b]	0.008	0.008	0.008	0.008	0.008	0.008	0.008
	p-value[c]	0.018	0.018	0.018	0.018	0.018	0.018	0.018
Phase	GVS	30.50±4.06	22.12±5.20	17.67±3.45	10.84±4.80	7.04±5.14	4.00±2.28	15.36±1.72
	Non-GVS	40.09±7.07	35.56±7.48	25.18±3.82	24.20±3.19	15.74±6.86	10.64±5.31	25.24±1.55
	p-value[a]	0.008	0.008	0.001	<0.001	0.012	0.005	<0.001
	p-value[b]	0.008	0.021	0.011	0.139	0.678	0.110	0.011
	p-value[c]	0.018	0.018	0.018	0.018	0.018	0.018	0.018
Symmetry	GVS	6.69±1.78	2.91±1.77	0.55±0.21	0.47±0.48	0.60±0.39	0.56±0.23	1.96±0.42
	Non-GVS	15.32±2.40	12.58±3.03	6.81±2.42	2.81±1.57	2.14±1.40	2.22±1.85	6.98±1.27
	p-value[a]	<0.001	<0.001	<0.001	0.001	0.003	0.142	<0.001
	p-value[b]	0.008	0.011	0.953	0.594	0.374	0.859	0.011
	p-value[c]	0.018	0.018	0.018	0.018	0.018	0.063	0.018

The average values of the two groupswere compared by Mann-Whitney U tests. The VOR changes in each group were tested by Wilcoxon signed rank tests. [a]=statistics between the GVS and non-GVS groups by Mann-Whitney U test. [b]=statistics between baseline and the GVS groups by Wilcoxon signed rank test. [c]=statistics between the baseline and non-GVS groups by Wilcoxon signed rank test. VOR, vestibulo-ocular reflex; GVS, galvanic vestibular stimulation.

Supplementary Table S3. The VOR response to sinusoidal stimulation 7 days post UL in the GVS and the non-GVS groups compared to baseline.

		0.08 Hz	0.1Hz	0.16 Hz	0.32 Hz	0.64 Hz	1 Hz	Total
Gain	GVS	0.56±0.05	0.63±0.06	0.72±0.05	0.75±0.06	0.76±0.06	0.72±0.06	0.69±0.05
	Non-GVS	0.22±0.04	0.25±0.01	0.36±0.03	0.42±0.04	0.48±0.08	0.44±0.09	0.36±0.04
	p-value[a]	<0.001	<0.001	<0.001	<0.001	<0.001	<0.001	<0.001
	p-value[b]	0.008	0.008	0.008	0.008	0.008	0.008	0.008
	p-value[c]	0.018	0.018	0.018	0.018	0.018	0.018	0.018
Phase	GVS	27.90±5.49	20.45±2.72	12.90±2.43	8.92±2.73	4.72±2.22	2.71±1.44	12.93±1.04
	Non-GVS	38.20±6.99	31.35±7.86	24.97±5.96	19.49±6.13	10.68±5.82	12.16±6.10	22.81±3.73
	p-value[a]	0.008	0.001	0.003	0.001	0.031	0.001	<0.001
	p-value[b]	0.011	0.011	0.374	0.441	0.314	0.678	0.139
	p-value[c]	0.018	0.018	0.018	0.018	0.018	0.018	0.018
Symmetry	GVS	1.92±0.75	0.81±0.26	0.67±0.25	0.53±0.28	0.44±0.23	0.29±0.21	0.78±0.15
	Non-GVS	10.17±2.94	8.55±3.79	4.83±2.50	2.69±2.22	1.35±0.91	0.93±0.21	4.75±1.66
	p-value[a]	<0.001	<0.001	<0.001	0.031	0.042	0.001	<0.001
	p-value[b]	0.441	0.515	0.594	0.953	0.678	0.139	0.314
	p-value[c]	0.018	0.018	0.018	0.043	0.028	0.018	0.018

The average values of the two groupswere tested by Mann-Whitney U tests. The VOR changes in each group were tested by Wilcoxon signed rank tests. [a]=statistics between the GVS and non-GVS groups by Mann-Whitney U test. [b]=statistics between baseline and the GVS groups by Wilcoxon signed rank test. [c]=statistics between the baseline and non-GVS groups by Wilcoxon signed rank test. VOR, vestibulo-ocular reflex; GVS, galvanic vestibular stimulation.

Supplementary Table S4. The VOR response to sinusoidal stimulation 14 days post UL in the GVS and the non-GVS groups compared to baseline.

		0.08 Hz	0.1Hz	0.16 Hz	0.32 Hz	0.64 Hz	1 Hz	Total
Gain	GVS	0.60±0.03	0.70±0.03	0.83±0.03	0.83±0.04	0.85±0.03	0.77±0.02	0.76±0.02
	Non-GVS	0.37±0.07	0.45±0.03	0.55±0.07	0.61±0.07	0.65±0.05	0.62±0.03	0.54±0.04
	p-value[a]	<0.001	<0.001	<0.001	<0.001	<0.001	<0.001	<0.001
	p-value[b]	0.008	0.008	0.173	0.139	0.953	0.066	0.008
	p-value[c]	0.018	0.018	0.018	0.018	0.018	0.018	0.018
Phase	GVS	26.46±3.56	20.04±4.38	14.84±2.05	7.85±2.22	4.53±3.06	2.57±1.36	13.05±1.47
	Non-GVS	29.67±4.80	25.88±6.59	18.36±4.82	13.38±3.50	10.57±2.86	4.15±1.52	17.00±2.08
	p-value[a]	0.606	0.091	0.142	0.002	0.001	0.114	0.001
	p-value[b]	0.066	0.038	0.028	0.859	0.051	0.767	0.086
	p-value[c]	0.028	0.018	0.018	0.043	0.018	0.310	0.018
Symmetry	GVS	1.80±1.77	0.68±0.38	0.78±0.78	0.50±0.26	0.41±0.33	0.55±0.41	0.79±0.53
	Non-GVS	7.98±4.74	3.04±1.69	1.26±0.68	0.88±0.50	0.64±0.25	0.48±0.34	2.38±0.92
	p-value[a]	<0.001	<0.001	<0.001	0.071	0.681	0.758	<0.001
	p-value[b]	0.214	0.515	0.139	0.678	0.260	0.767	0.594
	p-value[c]	0.018	0.018	0.018	0.128	0.310	0.735	0.018

The average values of the two groups were tested by Mann-Whitney U tests. The VOR changes in each group were tested by Wilcoxon signed rank tests. [a]=statistics between the GVS and non-GVS groups by Mann-Whitney U test. [b]=statistics between baseline and the GVS groups by Wilcoxon signed rank test. [c]=statistics between the baseline and non-GVS groups by Wilcoxon signed rank test. VOR, vestibulo-ocular reflex; GVS, galvanic vestibular stimulation.
